# Safety of thalidomide and bevacizumab in patients with hereditary hemorrhagic telangiectasia

**DOI:** 10.1186/s13023-018-0982-4

**Published:** 2019-02-04

**Authors:** Elisabetta Buscarini, Luisa Maria Botella, Urban Geisthoff, Anette D. Kjeldsen, Hans Jurgen Mager, Fabio Pagella, Patrizia Suppressa, Roberto Zarrabeitia, Sophie Dupuis-Girod, Claire L. Shovlin, on behalf of VASCERN-HHT, Paolo Federici, Claudia Crocione

**Affiliations:** 1 VASCERN HHT Reference Center, ASST Maggiore Hospital, Crema, Italy; 2 Department of Cell and Molecular Medicine Centro de Investigaciones Biológicas, CSIC, U707 CIBERER, Madrid, Spain; 3 VASCERN HHT Reference Center, Essen University Hospital, Department of Otorhinolaryngology, University of Duisburg-Essen, Essen, Germany; 4 VASCERN HHT Reference Center, Odense Universitetshospital, Syddansk Universitet, Odense, Denmark; 5 VASCERN HHT Reference Center, St Antonius Ziekenhuis, Nieuwegein, Netherlands; 6 VASCERN HHT Reference Center, Unità Operativa Complessa di Otorinolaringoiatria, University of Pavia, IRCCS Policlinico San Matteo Foundation, Pavia, Italy; 7 VASCERN HHT Reference Center, Centro sovraziendale Malattie rare, “Frugoni” Internal Medicine Unit, University of Bari “A. Moro”, Bari, Italy; 8 HHT Unit, Hospital Sierrallana, Cantabria, Spain; 9 VASCERN HHT Reference Center, Genetic department, Hospices Civils de Lyon, Femme-Mère-Enfants Hospital, F-69677 Bron, France; 10 VASCERN HHT Reference Center, Hammersmith Hospital, Imperial College Healthcare NHS Trust, London, and Vascular Sciences, National Heart and Lung Institute, Imperial College London, London, UK

**Keywords:** Hereditary hemorrhagic telangiectasia, Bevacizumab, Thalidomide, Adverse event, Bleeding, Arteriovenous malformation, Epistaxis, Cardiac failure, Nosebleeds

## Abstract

**Background:**

Hereditary hemorrhagic telangiectasia (HHT) is a multisystemic inherited vascular dysplasia that leads to nosebleeds and visceral arteriovenous malformations (AVMs). Anti-angiogenic drugs thalidomide and bevacizumab have been increasingly used off-label with variable results. The HHT working group within the ERN for Rare Multisystemic Vascular Diseases (VASCERN), developed a questionnaire-based retrospective capture of adverse events (AEs) classified using the Common Terminology Criteria for Adverse Events.

**Results:**

Sixty-nine HHT patients received bevacizumab, 37 (50.6%) for high output cardiac failure/hepatic AVMs, and 32 (49.4%) for bleeding; the 69 patients received bevacizumab for a mean of 11 months for a total of 63.8 person/years treatment. 67 received thalidomide, all for epistaxis and/or gastrointestinal bleeding; they received thalidomide for a mean of 13.4 months/patient for a total of 75 person/years treatment. AEs were reported in 58 patients, 33 with bevacizumab, 37 with thalidomide. 32 grade 1–3 AEs related to bevacizumab were reported with an average incidence rate of 50 per 100 person-years. 34 grade 1–3 AEs related to thalidomide were reported with an average incidence rate of 45.3 per 100 person-years. Bevacizumab AEs were more common in females (27 AEs in 46 women) than males (6 in 23, *p* < 0.001). Thalidomide AEs occurred at more similar rates in males (25 AEs in 41 men, 60.9%) and females (12 in 26 (46.2%), but were more common in *ENG* patients (17 in 17) than in *ACVRL1* (14 in 34, *p* < 0.0001). For bevacizumab, the most common reports were of joint pains (7/69, 10%), headache (3/69, 4.4%) and proteinuria (2/69, 3%), and for thalidomide, peripheral neuropathy (12/67, 18%); drowsiness (8/67, 12%); and dizziness (6/67, 9%). Fatal adverse events were more common in males (*p* = 0.009), and in patients with *ENG* pathogenic variants (*p* = 0.012). One fatal AE was possibly related to bevacizumab (average incidence rate: 1.5 per 100 person-years); 3 fatal AEs were possibly related to thalidomide (average incidence rate: 4 per 100 person-years).

**Conclusions:**

With potential increase in use of Bevacizumab and Thalidomide in HHT patients, data presented support appropriate weighing of the toxicities which can arise in HHT settings and the practice recommendations for their prevention and management.

**Electronic supplementary material:**

The online version of this article (10.1186/s13023-018-0982-4) contains supplementary material, which is available to authorized users.

## Background

Hereditary haemorrhagic telangiectasia (HHT) is a multisystemic inherited vascular dysplasia that leads to nosebleeds, arteriovenous malformations (AVMs) in organs such as the lungs, liver and brain [1–3]. HHT is estimated to affect 85,000 European citizens [3]. Unfortunately, most health care providers have limited specific knowledge, whereas dedicated competence is critical to deal with this rare disorder [3–8].

The clinical criteria for diagnosing HHT, the Curaçao criteria, were established by a panel of experts [1]. Most HHT patients have pathogenic variants in one of two known disease-related genes, *ENG* (endoglin, HHT1) or *ACVRL1* (activin A receptor type II-like 1, HHT2), which encode proteins involved in the transforming growth factor ß pathway [2]. Clinical presentation varies greatly depending on the number, type and location of telangiectases or AVMs with similar variation in potential morbidity and mortality. For example, one dominant clinical feature is iron deficiency anemia as a result of recurrent bleeds from either nasal or gastrointestinal telangiectases: these can lead to severe anemia requiring iron supplements and also recurrent need for blood transfusions. Other common manifestations, each present in approximately 50% of cases, are pulmonary and hepatic AVMs. Pulmonary AVMs provide direct communications between pulmonary arteries and veins (i.e. a right-to-left shunt) -the most important risks are paradoxical embolic strokes and brain abscess [6–8]. Hepatic AVMs unique to HHT involve the liver diffusely: intrahepatic shunting can lead to different clinical features, including high-output cardiac failure (HOCF), portal hypertension, encephalopathy, biliary ischemia, and mesenteric ischemia [4, 5].

Multiple approaches, including surgical options, have been tried in the management of HHT- related epistaxis or gastrointestinal bleeding. While most of them have variable and temporary results, there is randomized control trial evidence in HHT to support the use of tranexamic acid [9, 10] tamoxifen [11] and even simple topical nasal treatments such as saline sprays [12]. Such treatments and/or interventional procedures can often avoid the long term use of other drugs; however interventions can be associated with local complications such as perforation of the nasal septum, and drugs with other side effects, or limited individual response. As a consequence, most patients require repeated interventions and treatments, many with only partial responses.

In recent years, angiogenesis has been implicated in the pathogenesis of HHT, where circulating concentrations of both TGF-beta and vascular endothelial growth factor (VEGF) are significantly elevated [13]. Anti-angiogenic substances have been proposed as treatments for severe HHT-related bleeding, and for complicated hepatic AVMs. Both thalidomide (TH) and bevacizumab (BZB), have been increasingly used in the latest decade in HHT patients, within and outside expert HHT-centers.

BZB and TH use in oncological conditions is well established. TH is a potent immunosuppressive and antiangiogenic agent, [14–16] effective in the treatment of inflammatory diseases [17, 18], and in various cancers where VEGF plays an important role in tumor growth, invasion, and metastasis by promoting tumor angiogenesis [19–21]. Reduced bleeding has been observed in HHT patients who received TH as an antiangiogenic cancer therapy [22, 23]; TH treatment induced vessel maturation in an experimental model of HHT and reduced severe nosebleeds in six of the seven HHT patients studied [24]; and substantial improvements have been described in patients with other non HHT intestinal angiodysplasias treated with TH, when cessation of bleeding was associated with a reduction in serum VEGF levels [25, 26]. In a few small studies in HHT, TH consistently improved severity and frequency of epistaxis and improve hemoglobin concentrations while decreasing the need for transfusion [28–30]. Similarly, there is evidence for the efficacy of BZB in HHT. This humanized monoclonal antibody against VEGF, is approved in combination with chemotherapy for treating many types of advanced cancer, including colorectal cancer, non–small cell lung cancer, breast cancer, renal cell carcinoma, and glioblastoma multiforme [31, 32]. BZB improves anemia from chronic HHT-related bleeding [33, 34], and high cardiac output secondary to hepatic AVMs [35], in some cases, reversing the need for liver transplantation [36, 37].

To date, in HHT, the main indications for BZB and TH have been in two groups: (A) Patients with severe epistaxis, gastrointestinal bleeding or a combination of the two; and (B) for HOCF secondary to hepatic AVMs; either BZB or TH can be proposed for the first group of indications, whereas BZB only can be proposed for the second [22–38]. The use of BZB or TH is generally proposed when these severe HHT complications are refractory to other, often multiple, therapeutic attempts, which vary depending on type of complication; these severe HHT complications are frequently associated with substantial transfusion requirement [24, 28, 33–35]. So far, no data were systematically collected on safety profile of BZB and TH in HHT patients; it is not clear if there could be a different safety profile in HHT patients compared to oncological setting, where it has been shown that both TH and BZB expose patients to the risk of severe side effects [39–43]. On the other hand, evaluation of safety of BZB and TH in HHT settings is very important: 1) Theoretically, their antiangiogenetic actions on the perturbed vascular morphogenesis characteristic of HHT could result in either helpful vessel maturation [24] or in further bleeding and vascular complications [44–46]; 2) The use of BZB and TH in HHT is off-label and reported experience is still very limited; 3) These two drugs, which can represent the last resort for extremely sick patients and with compromised quality of life, could be used long term or even life-long.

In 2016 the European Commission launched the European Reference Networks (ERN), which are virtual networks involving healthcare providers across Europe, to tackle complex or rare diseases and conditions that require highly specialized treatment and a concentration of knowledge and resources. Presently, there are 24 ERNs involving 26 European countries, covering all major disease groups [47]. VASCERN-HHT comprises eight HHT reference centers (from UK, France, Italy [3 centers], Netherlands, Denmark, and Germany) [48].

A core recommendation of the ERN auditing authority adopted by the ERN for Rare Multisystemic Vascular Diseases (VASCERN) [49], was to foster information on safety standards for rare disease patients. The VASCERN working group dedicated to HHT prioritized anti-angiogenic agents TH and BZB in HHT, as both have a potential for adverse events that warrant a great level of attention from scientific, clinical and lay HHT communities. The aim of this study was to evaluate the safety of BZB and TH use in HHT patients treated within HHT expert centers.

## Methods

### Drug registry- part 1

A VASCERN-HHT Survey (Drug Registry- Part 1) was proposed first, to include all VASCERN-HHT stakeholders, including patient representatives and scientists, in addition to health care professionals (HCPs) working within, or in association with, the VASCERN HHT Centers. The HHT Centers involved in the study are tertiary care reference Centers for HHT, with similar case mixes of HHT patients, with an average HHT-specific experience of 21 years (range 15–27 years). The wide range of stakeholders consulted in Part 1 was designed to foster patient and scientific involvement with drug safety issues; cement the group’s interest by generating an early output [50] and encourage clinicians in the more demanding second stage that would be required.

The questionnaire is supplied in the Data Supplement (Additional file 1). Briefly, all respondents were asked to summarize their experience, if any, with these drugs, via an online questionnaire that included 4 questions dedicated to patients, 2 to scientists, and 13 to individual HHT Centers (the 8 in VASCERN and one cooperating Center). The questions focused on direct or indirect experience with BZB and TH, broad ranges of treated patients, and subjective agreements with a number of statements indicating perceived efficacy and safety of the two drugs in HHT. All responses were received between February 1st and 20th 2017.

In this questionnaire, responses were on a 1–7 scale where 1–3 represented disagreement (1 strongly, 2 with major reservation; 3 with minor reservation; 4 was don’t know, and 5–7 represented agreement: 5 with major reservation; 6 with minor reservation; 7 agree strongly). For graphical representation the scores were converted to − 3 to + 3 where 0 represented don’t know, 1–3 represented agreement (1 with major reservation; 2 with minor reservation; 3 agree strongly), and − 3 to − 1 represented disagreement (− 3 disagree strongly, − 2 disagree with major reservation; − 1 disagree with minor reservation).

### Drug registry- part 2

After evaluation of Part 1 responses [50] a second survey, Drug Registry-Part 2 was proposed in May 2017 only to clinicians in the HHT Centers, to formally capture any adverse events that may have occurred while patients were being treated with BZB and TH.

All HHT Centers submitted patients treated with these agents to regular follow up according to their established center protocols for HHT treatment and surveillance, including recording of periodical checks depending on different treatment schedules, and intercurrent events, as previously reported [28, 29, 35, 51]. Data provided by HHT Centers were obtained from their HHT-specific electronic health records.

Only one respondent was allowed to respond per HHT Center, and all replies were received between May 1st and July 15th 2017. The questionnaire of Drug Registry-Part 2, (supplied as Additional file 2), included 35 questions divided into 2 sections.

### Description of experience

The first questions were dedicated to description of the HHT Centers’ experience with either BZB or TH.

For each agent, Centers were asked for the number, age, sex, and genotype (*ENG*, *ACVRL1, SMAD4,* or unknown) of HHT patients; and specific indication for treatment. Treatment options provided were: otherwise untreatable epistaxis (nosebleeds), otherwise untreatable gastrointestinal bleeding, a combination of both nasal and gastrointestinal bleeding, or otherwise untreatable high output cardiac failure (HOCF)) for each patient. Further questions in this section were drug specific:

For TH, additional questions referred to the treatment duration expressed in months of therapy, the daily drug dosage (e.g. 50, 100, 200 mg), and the number of patients.

For BZB, additional questions referred to the treatment duration; number of patients treated with only an induction cycle (with 6 administrations every 2–3 weeks); number of patients treated with induction and maintenance; the total number of drug administrations; drug dosage (2.5 or 5 mg/kg and number of drug administrations); and details of the administration schedule (e.g. weeks’ interval for induction and for maintenance).

### Adverse events

The second section captured data on Adverse Events (AEs) using the common terminology criteria for adverse events (CTCAE, version 4.03, June 14, 2010) [52]. These define an Adverse Event (AE) as any unfavorable and unintended sign (including an abnormal laboratory finding), symptom, or disease temporally associated with the use of the medical treatment that may or may not be considered related to the medical treatment or procedure.

In the CTCAE, the severity of each AE is graded from 1 to 5 as follows: Grade 1 represents MILD, i.e. asymptomatic or mild symptoms, clinical or diagnostic observations only, where intervention not indicated. Grade 2 represents MODERATE, i.e. limiting age-appropriate instrumental activities of daily living (ADL), ie preparing meals, shopping for groceries or clothes, using the telephone, managing money, etc., and where minimal, local or noninvasive intervention is indicated. Grade 3 represents SEVERE, i.e. medically significant but not immediately life-threatening events such as hospitalization or prolongation of hospitalization; disabling symptoms; and symptoms limiting self-care such as bathing, dressing and undressing, feeding, using the toilet, and taking medications, but not bedridden. Grade 4 represents LIFE-THREATENING consequences, where urgent intervention is indicated. Grade 5 represents DEATH related to the AE.

Section 2 of the questionnaire Drug Registry-Part 2 was dedicated to the description of AE according system organ class (SOC) of CTCAE manual, identifying by anatomical or physiological system, etiology, or purpose (e.g., investigations for laboratory test results); within each system organ class, AEs were to be listed and accompanied by descriptions of severity (grade). A link to the CTCAE manual was provided within the questionnaire [52]. Every single AE that was not present before the treatment had to be described; the questionnaire allowed the description of multiple AE for the same patient.

A list of more common AE occurring with either TH or BZB was provided (systemic hypertension; gastrointestinal perforation; arterial thrombosis; venous thrombosis/thromboembolic event; cardiac failure; peripheral neuropathy; joint pain; bleeding or other (to be described). If bleeding, the site was to be specified between cerebral, pulmonary, gastrointestinal and other.

For every individual AE, further information was requested on the patient demographics; genotype; drug used (either BZB or TH); AE occurring either on treatment (number of months from treatment start), or off treatment (number of months since treatment stopped); drug dosage; AE type; and AE grade from 1 to 5 (death). For Grade 5 AE, it was to be specified if death was certainly related to the drug; whether the drug may have contributed; whether the death was not related to the drug; or if causality status was unknown. Details of the AE outcome were requested (resolved completely, resolved with sequelae, unresolved/worsened, or unknown); if the treatment was interrupted or not because of the AE (and if yes, whether the AE improved after treatment interruption); eventual treatment restart or not (and if yes whether an AE that had improved recurred or not after treatment restart); any use of any other concomitant drugs possibly related to the AE. For fatal AEs, further case details were requested from the HCP.

The questionnaire-based survey was approved by the Ethics Committee of Maggiore Hospital ASST Crema, Italy.

### Data analyses

STATA IC 15 (StataCorp, Texas) and GraphPad Prism 5 (Graph Pad Software Inc., San Diego) were used to calculate distributions of variables, to perform comparisons between groups, and to generate graphs. Two group comparisons were by Mann Whitney rank for continuous data or chi squared/Fisher’s exact test for categorical data.

## Results

### Drug registry- part 1: Estimations of efficacy and safety

There were 15 respondents for Drug Registry-Part 1 from European HHT Centers, and additional responses from patient representatives (*N* = 2) and HHT scientists (*N* = 3). No patient representative had experience of either BZB or TH. Two scientists reported awareness of effects of BZB or TH in HHT patients. For TH, two individual HHT Centers reported experience with 20–50 patients, one with 6–20 patients and two with fewer than 5 patients. For BZB, one individual HHT Center reported experience with 20–50 patients, one with 6–20 patients and four with fewer than 5 patients.

**Fig. 1:**
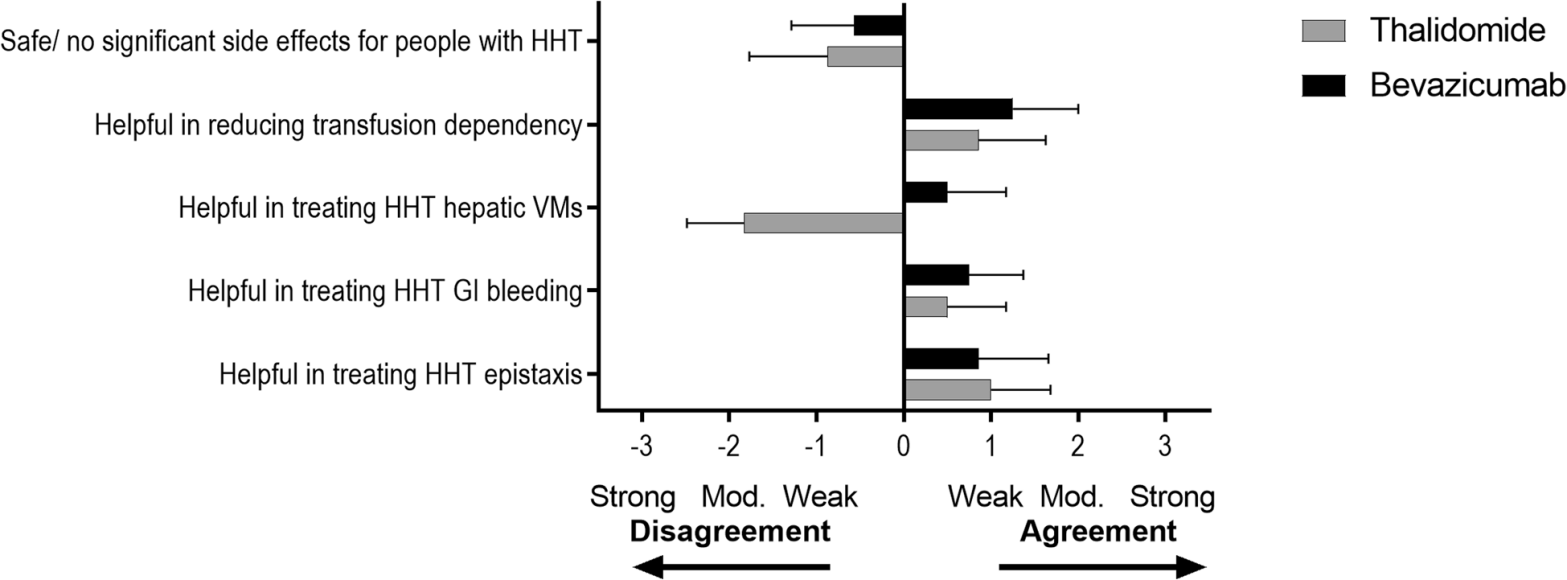
Drug Registry-part 1: estimations of efficacy and safety are represented in panel labels:. Mean agreement with statement where 0 represented don’t know, 1–3 represented agreement (1 with major reservation; 2 with minor reservation; 3 agree strongly), and − 3 to − 1 represented disagreement (− 3 disagree strongly, − 2 disagree with major reservation; − 1 disagree with minor reservation).

As noted in Fig. 1, there was agreement (with major reservations) that both TH and BZB could be helpful in treating HHT-related bleeding, and that BZB was helpful to treat hepatic AVMs. There was disagreement (with minor-major reservation) that the drugs were safe with no significant side effects for people with HHT.

### Drug registry-part 2: Adverse event evaluation

To examine further, the responses to the Drug Registry Part 2 were evaluated. Eight European HHT Centers had recommended or prescribed at least one of the drugs; six centers had prescribed BZB, four had prescribed TH. The average enrollment start for either BZB or TH was 6.5 years before the presented survey (range 3–9 years).

In total these HCPs reported 67 patients (mean age 66.4ys) treated with TH, and 69 (mean age 63.6ys) with BZB. 91 (66.9%) had pathogenic variants in *ACVRL1 (*HHT type 2); 27 (19.9%) in *ENG*, and 3 (2.2%) in *SMAD4.* Each drug was prescribed for a variety of indications, most commonly high output cardiac states for BZB, and epistaxis for TH. Table 1 summarises the characteristics of patients, and indications for treatment with either BZB or TH.

**Table 1:** Demographics and indications for treatment

	Thalidomide (*N* = 67)		Bevacizumab (*N* = 69)	
	N	%	N	%
Gender (% male)	41	61.2	23	33.3
Pathogenic gene				
*ENG *(HHT1)	17	25.4	10	14.5
*ACVRL1 *(HHT2)	34	50.7	57	82.6
*SMAD4* (JPHT)	2	3.0	1	1.4
Not known	14	20.9	1	1.4
Treatment indication				
Epistaxis	48	71.6	14	20.3
GI bleeding	11	16.4	8	11.6
Epistaxis and GI bleeding	8	11.9	10	14.5
High output cardiac failure	0	0	37	53.6

Legend: *N* number of cases treated, *GI* gastrointestinal

### Treatment schedules

The reported treatment schedules with dosages used are provided in Table 2. These varied for both drugs. The induction regimen for BZB consisted of 6 administrations in all 69 treated patients: most BZB schedules were of an induction regime with 5 mg/kg every 2–3 weeks, followed by a maintenance dose of 5 mg/kg every 4–12 weeks.

**Table 2:** Treatment schedules

A) BZB treatment Number of patients (number of drug administrations)		Treatment schedule		Number of drug administrations for different dosages		
Induction only	Induction + maintenance	Induction schedule (HCP number)	Maintenance range	5 mg/kg	2.5 mg/kg	1 mg/kg
38 (228)	31 (481)	Every 3 wks (3) Every 2 wks (3)	Every 4–12 weeks	669	4	36
B) TH treatment Months of treatment in 67 patients		Pts with daily dosage < 50 mg	Pts with daily dosage 50 mg	Pts with daily dosage 100 mg	Pts with daily dosage 200 mg	Pts/Mean daily dosage^a^
900		31	26	2	2	5/90 1/150

Legend: ^a^in patients treated with different dosages along the treatment

TH dosages were evenly split between < 50 and > 50 mg/day, with occasional patients receiving 100 mg/day or 200 mg/day.

### Treatment duration

For BZH, 38/69 patients had an induction only regimen, with a mean induction duration of 3.1 months/patient accounting for a total treatment duration of 120.1 months across all patients. 31/69 patients had both induction and maintenance, with a mean treatment duration of 20.8 months/patient, accounting for a total treatment duration of 646.4 months across all patients. Altogether, the 69 patients were treated with BZB for a mean period of 11 months providing data on a total of 63.8 person/years treatment.

The 67 patients receiving TH were treated for a mean period of 13.4 months/patient providing data on a total of 75 person/years treatment.

### Adverse events

Table 3 provides a summary of the AEs that occurred with either BZB or TH, with timing of AEs according to different AE grade. AEs Grade 1–5 were observed although no AE grade 4 was observed. No off-treatment AEs were reported.

**Table 3:** Summary of events

	AE grade	Number (% of AE)	Sex, male N	Age, mean (range)	Ratio *ENG: ACVRL1: SMAD4:* not known	Dose, mg/kg	Dose, mg/day, mean (range)	Number of patients	Mean months of treatment at AE
BZB	1	5 (15)	2	65 (61–69)	1:3:0:1	5		5	44.8
	2	17 (51)	1	60.3 (35–72)	0:9:1:7	5		10	45.6^a^
	3	10 (30)	2	56.9 (54–73)	0:5:0:5	5		5	36.8^b^
	4	0	–	–	–	–	–	–	–
	5	1 (3)	1	67	1:0:0:0	5	–	1	36
TH	1	8 (21)	5	67 (50–90)	2:5:0:0	–	75 (25–100)	7	41.5
	2	20 (54)	15	64.2 (53–81)	9:6:1:0		98 (50–200)	16	36.8
	3	6 (16)	2	65.8 (59–69)	4:2:0:0		75 (50–100)	6	23.5
	4	0	–	–	–	–	–	–	–
	5	3 (8)	3	66.3 (62–78)	2:1:0:0	–	100 (50–200)	3	11.6

Legend: ^a^not available in 7; ^b^not available in 5

Seventy AE were reported, 33 in 28 patients treated with BZB (average 1.18 events per patient), 37 in 30 patients treated with TH (average 1.23 events per patient).

Table 4 provides a summary of proportion of AEs according to patient sex and genotype. Women were more likely to have reported AEs with BZB (27 AEs in 46 women [58.7%]) compared to 6 AEs in 23 men [26.1%], *p* < 0.001, although the only fatal event occurred in a male. For TH, slightly more men reported AEs (25 AEs in 41 men [60.9%]) compared to 12 AEs in 26 women [46.1%], but the difference was not statistically significant *p* = 0.32. There was no evident genotypic difference in AE reports in patients using BZB, but there was a trend for *ENG* patients to have more AEs reported when on TH (17 in 17 *ENG* patients compared to 14 in 34 *ACVRL1* patients, *p* < 0.001).

**Table 4:** Proportion of AEs according to patients’ sex and genotype

	TH			BZB		
	TH treatment	TH AE	% of pts with AE	BZB treatment	BZB AE	% of pts with AE
male pts	41	25	61	23	6	26
female pts	26	12	46^a^	46	27	59^b^
*ENG* pts	17	17	100	10	2	20^c^
ACVRL1 pts	34	14	41	57	17	30^d^

Comparing males to females : ^a^*p*=0.32, ^b^<0.01. Comparing thalidomide to bevacizumab, ^c^*p*<0.001, d=0.36

41/69 (59%) users of BZB and 37/67 (55%) users of TH had no AEs.

The trends for number and severity of AEs with either BZB and TH across grades 1–5 did not reach statistical significance (*p* value > 0.1). However, AEs tended to occur earlier with thalidomide than with BZH, with 14/34 (41.2%) versus 5/32 (16%) occurring within first 6 months of treatment (*p* value = 0.030).

### AEs grades 1–3

Table 5 shows AE type, classified by organ system, and outcomes for AEs grade 1–3.

**Table 5:** AE type and outcomes for AEs grade 1–3

	AE grade	System Organ Class (SOC)/AE type								AE outcome	Treatment interruption	Improvement after interr	Treatment restart	Alternative cause for AE
		General disorders	Vascular disorders	Musculo-Skeletal disorders	Nervous System & Psychiatric disorders	Renal disorders	Gastroint. disorders	Reproduct system disorders	Skin disorders					
BZB	1		1 enlarged PAVM	1 joint pain 1 facial pain					2 hair loss	1 unresolved/worsened 4 resolved	1 Yes 4 No	1 No	1 No	In 2
	2	1 fatigue	2 hypertension	6 joint pain 1 vertebral fracture	1 confusion 3 headaches	2 proteinuria	1 diarrhea			17 resolved	17 No			In 1
	3	1 fatigue	3 hypertension 1 arterial thrombosis^a^	2 joint pain	1 confusion		1 GI bleeding^b^ 1 diarrhea			1 unresolved/worsened 9 resolved	3 Yes 7 No	2 Yes 1 No	1 Yes 2 No	In 2
TH	1				5 peripheral neuropathy^c^ 1 somnolence 1 drowsiness				1 rash	2 unresolved/worsened 6 resolved	3 Yes 5 No	3 Yes	3 No	none
	2				5 peripheral neuropathy^d^ 4 dizziness 2 somnolence 7 drowsiness		1 constipation	1 erectile dysfunction		7 unresolved/worsened 2 resolved with sequelae 11 resolved	15 Yes 5 No	10 Yes 5 No	15 No	none
	3	1 limb edema	1 arterial thrombosis^f^		2 peripheral neuropathy & 2 dizziness					4 unresolved/worsened 2 resolved	4 Yes 2 No	2 Yes 2 No	1 Yes 3 No	In 1

Legend: ^a^unresolved/worsened after treatment interruption

^b^resolved completely

^c^3 resolved completely, 2 unresolved/worsened,

^d^1 resolved completely, 1 resolved with sequelae, 3 unresolved/worsened

^&^2 unresolved/worsened

^f^resolved completely

Thirty two grade 1–3 AEs related to BZB were reported with an average incidence rate of 50 per 100 person-years. Thirty four grade 1–3 AEs related to TH were reported with an average incidence rate of 45.3 per 100 person-years.

For BZB, the most common reports were of joint pain (9/69, 13%) and hypertension (5/69, 7.2%);

For TH, the most common reports were of peripheral neuropathy (12/67, 18%), drowsiness (8/67, 12%), and dizziness (6/67, 9%). Somnolence and drowsiness were all grade 1–2, required treatment interruption in 4 cases (in one grade 1 and three grade 2) and resolved completely after treatment interruption. Peripheral neuropathy with TH remained unresolved or worsened in two thirds of cases of present study.

### AEs grade 5

Death occurred in 4 patients (all men) while on treatment: three had *ENG* pathogenic variants and 1 had *ACVRL1.* Across the full population of treated patients, this excess in *ENG* deaths was statistically significant (*p* = 0.017).

As noted in Table 6, there was one fatal AE on BZB treatment. This was a 67-year-old male who had tolerated effective BZB treatment for 65 months (maintenance dose 5 mg/kg BZB infusion every 2 months). A mild hemoptysis at month 65 resulted in a thoracic CT scan that demonstrated pulmonary bleeding from a pulmonary AVM. The patient died from a catastrophic hemoptysis while waiting for urgent pulmonary AVM embolization. The AE was deemed possibly related to drug; pulmonary AVM spontaneous rupture was considered an alternative cause for patient death, noting such an event is highly unusual outside of pregnancy or pulmonary hypertension, which the patient did not have. The fatal AE was considered possibly related to BZB with an average incidence rate of 1.5 per 100 person-years.

**Table 6:** AE type and outcomes for AEs grade 5 (fatal)

	Age	Sex	Gene	Indication for treatment			Pulmonary AVMs	Treatment response	Treatment duration at event	Fatal event (SOC)	Drug-related AE?
				Bleeding	Support	Mean Hemoglobin (Hb)					
BZB	67	Male	*ENG*	Refractory GI bleeding	Regular blood transfusions	< 6 g/dL	Previously treated, no follow up for 5 ys	Excellent, mean Hb > 10 g/dL	65 months	Haemoptysis from ruptured PAVM (Vasc Disord)	Possibly related ; theoretical alternate cause: spontaneous rupture
TH	69	Male	*ACVRL1*	Refractory epistaxis	Regular blood transfusions	< 7 g/dL	no	Good: mean Hb > 9 g/dL; fewer tx	10 months	Cardiac failure (Card Disord)	Possibly related; theoretical alternate cause: ischemic cardiopathy
	62	Male	*ENG*	Refractory GI bleeding	Regular blood transfusions	< 7 g/dL	no	Partial, mean Hb > 8 g/dL; fewer tx	23 months	Ischemic stroke (Vasc Disord)	possibly related; theoretical alternate cause: atherosclerosis
	78	Male	*ENG*	Refractory epistaxis	Regular blood transfusions	< 7 g/dL	no	Good: mean Hb > 9 g/dL; fewer tx	2 months	Catastrophic epistaxis (Resp Disord)	possibly related; theoretical alternate cause: spontaneous nosebleed

Legend: Drug dosing schedules provided in the text. *SOC* System Organ Classification

There were three fatal AEs during TH treatment (Table 6). All 3 cases had tolerated treatment for 1–23 months before the AE. The first, a 69-year-old male had tolerated effective treatment with TH 50 mg/day until month 10 of treatment, when he died of cardiac failure. The AE was deemed possibly related to the drug. Ischemic cardiopathy was considered an alternative cause of patient death although there was no evidence for this*.* The second, **a** 62-year-old male had tolerated treatment with TH 200 mg/day that was partially effective. On month 23 of treatment patient died of ischaemic stroke. The AE was deemed possibly related to the drug; atherosclerosis was considered an alternative cause of patient death. The third, a 78-year-old male had tolerated treatment with TH at 50 mg/day for 1 month. The dose was increased to 100 mg/day and initially tolerated and effective, but on month 2 of treatment patient died of a catastrophic nosebleed. The AE was deemed possibly related to drug; a spontaneous catastrophic nosebleed was considered an alternative cause for patient death. The three fatal AEs were considered possibly related to TH with an average incidence rate of 4 per 100 person-years.

## Discussion

The focus of our study is on the occurrence of adverse events during BZB and TH treatment of HHT-related manifestations. Both drugs are associated with adverse events at respective event rates of 0.40 and 0.44 AEs per patient.

The strength of the present study is the evaluation of safety of BZB and TH in HHT within expert HHT centers which can offer a specific disease knowledge, an established surveillance schedule and an appropriate indication for the use of these drugs; furthermore, data provided by the present survey on safety profiles of BZB and TH in HHT patients can assist therapeutic decisions by an appropriate risk weighing, and this is particularly important in HHT complications which generally require long term treatments. Study limitations include the fact that the data were collected from Centers of reference for HHT: it has to be underscored that within European HHT Reference Centers, antiangiogenic drugs are generally reserved for patients with severe conditions (either nose or gastrointestinal bleeding, or high output cardiac decompensation) and refractory to other therapies. In such a context of critically ill patients it could be difficult to discriminate if a serious event is related to either the disease or to the drug. Retrospective collection of data may have entailed underreporting and underestimation of AEs; however within HHT Centers of reference patients are followed up periodically, particularly if on particular treatments as BZB/TH, and patients are instructed to report to the Center any problem they may encounter. This surveillance policy should have limited the possibility of missing AEs.

The size of treatment groups may seem modest but it should be remembered that the potential study cohort are a small subgroup of patients within a rare disease. In the October 2018 VASCERN Meeting, the clinical experts within VASCERN HHT estimated that the patients with HHT complications sufficiently severe to warrant BZB or TH represent fewer than 5% of HHT patients seen by them [49, 53]. Thus, based on 85,000 prevalent HHT cases in Europe, fewer than 4250 would be expected to have a severe presentation, refractory to first line treatments, possibly with substantial transfusion requirement. Similarly, within the current European population of 512 millions [54], the proportion of severe HHT cases in Europe would be in the order of 8/ million inhabitants. In support, the centers of reference of VASCERN HHT participating to this study treated with BZB/TH an average of 2.6 patients/per year/per center (the Danish Center, which has a 70% recruitment of HHT cases from the country, treated an average of 2.5 patients/year, over a Danish population of 5,770,000 (personal communication)).

It has to be emphasized, as it is usual for rare conditions, that dedicated competence is critical to evaluate and to treat HHT and two of its worst presentations of severe transfusion-dependent anemia due to chronic bleeding, and/or or high output cardiac failure due to liver AVMs. As part of the advice of an HHT center of reference, potential risks are weighed together with potential benefits in terms of survival and quality of life conferred by the addition of BZB/TH in critically ill HHT patients. Additionally, the experienced HHT centers of reference are aware of the full and emerging spectrum of HHT, [3, 5–10, 28, 35, 50, 51, 53, 55–66], and therapeutic options to offer the best care possible, including the use of other drugs and/or interventional procedures which can often avoid the use of BZB/TH. In the present study there was not a particular Center propensity for either BZB or TH, and the drug choice depended on previous experiences [28, 29, 35, 51] rather than on different patient recruitment.

Toxicities related to the use of BZB and TH are well recognized due to experience in oncological use. Teratogenicity is the most serious side effect of antiangiogenic drugs. TH and BZB are absolutely contraindicated in women who are, or could become, pregnant. Very common AE (> 10%) reported in relation to TH include constipation, leukopenia, anemia, thrombocytopenia, peripheral neuropathy, dizziness, impotence, thyroid dysfunction, and edema. Less common AE (1–10%) of TH include heart failure, deep vein thrombosis and pulmonary embolism [39]. Toxicities are also encountered with BZB use, in particular grade 3 medically-manageable hypertension (3–16%). In addition, other serious and sometimes fatal AEs are hemorrhage, gastrointestinal perforation related to tumor necrosis, thromboembolic events, wound healing complications, neutropenia, and nephrotic syndrome [40–43]. Table 7 illustrates the comparison of rates of main AEs reported in the present study in treating HHT patients and relevant figures reported in other settings. Whereas the rates of main AEs appear similar in these different settings, it is to be noted that bleeding complications are not reported in the use of TH outside the HHT condition; that the common presence of pulmonary AVMs in HHT renders likely paradoxical embolism of venous thromboemboli; that HHT patients can already have a high output cardiac state that enhances risks of cardiac compromise; and that in contrast to oncological conditions, HHT is not a particularly life-limiting condition [65, 66].

**Table 7:** Main AEs’ rates (for grades 1–5) in present series compared to literature data on BZB and TH in oncology (or other) settings

	Present study %	Literature [64–72] %
Bevacizumab		
Hypertension	7.2	5–19
Bleeding	2.8	1.7–6.7
Proteinuria	2.8	0.7–7.4
Arterial thromboembolism	1.4	0.7–4.4
Peripheral neuropathy	Not described	6.3
Thalidomide		
Somnolence/drowsiness	16	2–23
Peripheral neuropathy	17.9	1–44
Thromboembolic event	2.9	1–6
Cardiac failure	1.4	1–8
Bleeding	1.4	Not described

The current study has shown that AEs of lesser grade (1–2) were common, and, not surprisingly, included somnolence and drowsiness, typical of the sedative properties of TH. These minor side effects are however important for patients, as they affect quality of life of patients who need long term treatment, and as noted, can be the reason for treatment interruption. A non negligible rate of AE grade 2–3 were reported for both drugs (39%), with joint pain and peripheral neuropathy being the most frequent grade 1–3 AE for BZB and TH respectively. Peripheral neuropathy is a common, potentially severe side effect that, as suggested by the current data, may be irreversible with TH. It is a dose–dependent AE and generally occurs following chronic use over a period of months; however, reports following relatively short-term use also exist. Clinical assessment for symptoms and signs of peripheral neuropathy should be performed prior to and during TH treatment. If grade 1 or grade 2 neuropathy, the dose can be reduced by up to 50% of the last dose, whereas in the event of grade 3 or 4 neuropathy, treatment should be discontinued.

Arterial thrombosis complicated 1% of either BZB or TH treatments, and in the case occurring during BZB treatment, worsened even when treatment was interrupted. One of 4 fatal AEs was due to an ischemic stroke occurring during TH treatment. Thromboembolic events are well known AEs of both BZB and TH; patients with HHT receiving BZB may develop systemic or deep vein thrombosis [44, 45].

Bleeding events occurred in 2 patients (3%) treated with BZB, as grade 3 GI bleeding in 1 case and as catastrophic fatal pulmonary hemorrhage in 1 case; 1 case of catastrophic fatal nose bleeding occurred in 1 patient (1%) treated with TH. All three AEs were deemed possibly drug related. What is peculiar of two fatal bleedings that occurred in the present study is the catastrophic character of hemorrhage which did not leave time for any treatment. This is unusual in HHT: nosebleeds are almost never life-threatening events, even in patients anticoagulated with warfarin [61]. Furthermore, pulmonary AVMs very rarely rupture, with almost all fatal events described in the setting of pregnancy, pulmonary hypertension, excessive anticoagulation, or thrombolysis [6, 60, 62]. In the present study, a 68 year old patient treated with BZB presented enlargement of a pulmonary AVM at 24 months of treatment, which is again unusual in HHT outside of pregnancy and at this age [6, 63]. Catastrophic fatal GI bleeding, which again is not the rule for GI bleeding in HHT [64], has been reported in a patient treated with BZB for complicated liver AVMs [46]. These data could suggest a potential role of antiangiogenic drugs in destabilization and/or growth of AVMs in HHT.

Some suggestions can be drawn from these data on AEs of BZB/TH in HHT, and particularly on vascular complications:

i). In severe HHT-related conditions specific expertise on HHT is required to appropriately weigh benefits and risks of different available treatments [3–6];
ii). The present study has shown broadly similar potentials for AEs for BZB and TH. Therefore, for refractory HHT bleeding where either BZB or TH can be proposed, after a careful evaluation of risk-benefit balance on an individual basis, the two drugs can be equally considered for patients. However, the subgroup analyses suggest that *ENG* patients (both genders) may be more prone to AEs on TH, and that females may be more prone to AEs when on BZH. Therefore, when discussing potential risks of treatment, clinicians may include the possibility that due to a specific gender/HHT genotype combination, an individual patient may be more prone to side effects from a particular agent. Until further data on relative efficacies or AEs by gender, genotype or other patient subtype emerge, where other indications are comparable, clinicians may prefer to direct males with *ENG* variants to BZH rather than TH, and females with non *ENG* variants to TH rather than BZH. Other genotype/gender combinations have one factor in favour of each drug and no preference can be suggested at present.
iii). Evaluation of prothrombotic conditions should be considered before treatment with BZB and TH is started. Patients at high risk for thromboembolic events should be excluded from these treatments.
iv). Screening and treatment of pulmonary AVMs according to current guidelines [3, 4, 6] should be performed for every patient with HHT and particularly before considering BZB or TH treatment;
v). As vascular complications can be asymptomatic [44, 73], there is a likely need for enhanced surveillance of pulmonary AVMs during BZB/TH treatment to check pulmonary AVMs size. Whether alternate strategies should be routinely employed to exclude deep vein thrombosis is not yet clear, but the possibility should be constantly considered.
vi). Even minimal hemoptysis in an HHT patient on an antiangiogenic drug should prompt intensive management with thoracic CT scan, bronchoscopy, and embolization of pulmonary AVMs if needed. Discontinuation of the antiangiogenic drug is mandatory in such settings.
vii). After a careful evaluation of cost-benefit balance, BZB represents an interesting option for patients with complicated liver AVMs, refractory to first-line treatment [53] and not amenable to OLT, either over the age of 65 years or poor candidates for surgery. If they respond to the drug, they should be re-evaluated for OLT with a“fast-track” to minimize the potential for AEs due to BZB use [5, 58, 59].

## Conclusions

This study evaluated the safety of BZB and TH in HHT within expert HHT centers which can offer a specific disease knowledge, an established surveillance schedule, and an appropriate indication for the use of these drugs. Importantly, to weigh against potential benefits, both BZB and TH expose patients to the risk of severe side effects, with respective event rates of 0.40 and 0.44 AEs per HHT patient, including fatalities.

With potential increase in use of BZB and TH in HHT patients, these data support appropriate weighing of the toxicities which can arise from these drugs and the practice recommendations for their prevention and management. The risk profile for TH to BZB resulting from the data generated is helpful to share in pre-treatment counselling.

## Additional files

Additional file 1: Questionnaire for VASCERN-HHT Survey Drug Registry- Part 1. (PDF 183 kb)

Additional file 2: Questionnaire for VASCERN-HHT Survey Drug Registry- Part 2. (PDF 239 kb)

## Abbreviations

ACVRL1: Gene encoding the ALK-1 protein
AE(s): Adverse Event/s
AVM(s): Arteriovenous malformation(s)
BZB: Bevacizumab
ENG: Gene encoding the endoglin protein
ERNs: European Reference Networks
HHT: Hereditary haemorrhagic telangiectasia;
HOCF: High-output cardiac failure
SMAD4: Gene encoding the SMAD4 protein
SOC: System Organ Classification
TH: Thalidomide
VASCERN: European Reference Network for Rare Vascular Diseases
VEGF: Vascular endothelial growth factor

## References

[1] Shovlin CL, Guttmacher AE, Buscarini E, Faughnan ME, Hyland RH, Westermann CJ (2000). Diagnostic criteria for hereditary hemorrhagic telangiectasia (Rendu-Osler-Weber syndrome). Am J Med Genet.

[2] McDonald J, Wooderchak-Donahue W, VanSant WC, Whitehead K, Stevenson DA, Bayrak-Toydemir P (2015). Hereditary hemorrhagic telangiectasia: genetics and molecular diagnostics in a new era. Front Genet.

[3] Shovlin CL, Buscarini E, Kjeldsen AD, Mager HJ, Sabba C, Droege F (2018). European reference network for rare vascular diseases (VASCERN) outcome measures for hereditary Haemorrhagic telangiectasia (HHT). Orphanet J Rare Dis.

[4] Faughnan ME, Palda VA, Garcia-Tsao G, Geisthoff UW, McDonald J, Proctor DD (2011). International guidelines for the diagnosis and management of hereditary haemorrhagic telangiectasia. J Med Genet.

[5] European Association for the Study of the Liver. Vascular diseases of the liver. Garcia-Pagàn JC, Buscarini E, Janssen HL, Leebeck FW, Plessier A, Rubbia Brandt L et al. J Hepatol 2016; 64:179–202. 10.1016/j.jhep.2015.07.040 26516032

[6] Shovlin CL, Condliffe R, Donaldson JW, Kiely DG, Wort SJ (2017). British Thoracic Society clinical statement on pulmonary arteriovenous malformations. Thorax.

[7] Dupuis-Girod S, Cottin V, Shovlin CL (2017). The lung in hereditary hemorrhagic telangiectasia. Respiration.

[8] Boother EJ, Brownlow S, Tighe HC, Bamford KB, Jackson JE, Shovlin CL (2017). Cerebral abscess associated with odontogenic bacteremias, hypoxemia, and iron loading in immunocompetent patients with right-to-left shunting through pulmonary arteriovenous malformations. Clin Infect Dis.

[9] Gaillard S, Dupuis-Girod S, Boutitie F, Rivière S, Morinière S, Hatron PY, ATERO study group (2014). Tranexamic acid for epistaxis in hereditary hemorrhagic telangiectasia patients: a European cross-over controlled trial in a rare disease. J Thromb Haemost.

[10] Geisthoff UW, Seyfert UT, Kübler M, Bieg B, Plinkert PK, König J (2014). Treatment of epistaxis in hereditary hemorrhagic telangiectasia with tranexamic acid - a double-blind placebo-controlled cross-over phase IIIB study. Thromb Res.

[11] Yaniv E, Preis M, Hadar T, Shvero J, Haddad M (2009). Antiestrogen therapy for hereditary hemorrhagic telangiectasia: a double-blind placebo-controlled clinical trial. Laryngoscope.

[12] Whitehead KJ, Sautter NB, McWilliams JP, Chakinala MM, Merlo CA, Johnson MH (2016). Effect of topical intranasal therapy on epistaxis frequency in patients with hereditary hemorrhagic telangiectasia: a randomized clinical trial. JAMA.

[13] Sadick H, Riedel F, Naim R, Goessler U, Hörmann K, Hafner M (2005). Patients with hereditary hemorrhagic telangiectasia have increased plasma levels of vascular endothelial growth factor and transforming growth factor-beta1 as well as high ALK1 tissue expression. Haematologica.

[14] Barnhill RL, Doll NJ, Millikan LE, Hastings RC (1984). Studies on the anti-inflammatory properties of thalidomide: effects on polymorphonuclear leukocytes and monocytes. J Am Acad Dermatol.

[15] Moreira AL, Sampaio EP, Zmuidzinas A, Frindt P, Smith KA, Kaplan G (1993). Thalidomide exerts its inhibitory action on TNF-by enhancing the mRNA degradation. J Exp Med.

[16] D’Amato RJ, Loughnan MS, Flynn E, Folkman J (1994). Thalidomide is an inhibitor of angiogenesis. Proc Natl Acad Sci U S A.

[17] Grinspan D (1985). Significant response of oral aphthosis to thalidomide treatment. J Am Acad Dermatol.

[18] Hamuryudan V, Mat C, Saip S, Ozyazgan Y, Siva A, Yurdakul S (1998). Thalidomide in the treatment of the mucocutaneous lesions of Behcet’s syndrome: a randomized, double-blind, placebo-controlled trial. Ann Intern Med.

[19] Barlogie B, Desikan R, Eddlemon P, Spencer T, Zeldis J, Munshi N (2001). Extended survival in advanced and refractory multiple myeloma after single-agent thalidomide: identification of prognostic factors in a phase 2 study of 169 patients. Blood.

[20] Singhal S, Mehta J, Desikan R, Ayers D, Roberson P, Eddlemon P (1999). Anti-tumor activity of thalidomide in refractory multiple myeloma. N Engl J Med.

[21] Palumbo A, Bringhen S, Caravita T, Merla E, Capparella V, Callea V, et al. Oral melphalan and prednisone chemotherapy plus thalidomide compared with melphalan and prednisone alone in elderly patients with multiple myeloma: randomised controlled trial. Lancet, 2006;367:825. 10.1016/S0140-6736(06)68338-4 16530576

[22] Kurstin R (2002). Using thalidomide in a patient with epithelioid leiomyosarcoma and Osler-weber-Rendu disease. Oncology.

[23] Pérez-Encinas M, Rabunal Martinez MJ, Bello Lopez JL (2002). Is thalidomide effective for the treatment of gastrointestinal bleeding in hereditary hemorrhagic telangiectasia. Haematologica.

[24] Lebrin F, Srun S, Raymond K, Martin S, van den Brink S, Freitas C (2010). Thalidomide stimulates vessel maturation and reduces epistaxis in individuals with hereditary hemorrhagic telangiectasia. Nat Med.

[25] Kirkham SE, Lindley KJ, Elawad MA, Blanshard C, Shah N (2006). Treatment of multiple small bowel angiodysplasia causing severe life-threatening bleeding with thalidomide. J Pediatr Gastroenterol Nutr.

[26] Bauditz J, Lochs H, Voderholzer W (2006). Macroscopic appearance of intestinal angiodysplasias under antiangiogenic treatment with thalidomide. Endoscopy.

[27] Dabak V, Kuriakose P, Kamboj G, Shurafa M (2008). A pilot study of thalidomide in recurrent GI bleeding due to angiodysplasias. Dig Dis Sci.

[28] Hosman A, Westermann CJ, Snijder R, Disch F, Mummery CL, Mager JJ (2015). Follow-up of thalidomide treatment in patients with hereditary Haemorrhagic telangiectasia. Rhinology.

[29] Invernizzi R, Quaglia F, Klersy C, Pagella F, Ornati F, Chu F (2015). Efficacy and safety of thalidomide for the treatment of severe recurrent epistaxis in hereditary haemorrhagic telangiectasia: results of a non-randomised, single-center, phase 2 study. Lancet Haematol.

[30] Halderman AA, Ryan MW, Clark C, Sindwani R, Reh DD, Poetker DM, et al. Medical treatment of epistaxis in hereditary hemorrhagic telangiectasia: an evidence-based review. Int Forum Allergy Rhinol. 2018. 10.1002/alr.22094. 10.1002/alr.22094 29393992

[31] Hurwitz H, Fehrenbacher L, Novotny W, Cartwright T, Hainsworth J, Heim W (2004). Bevacizumab plus irinotecan, fluorouracil, and leucovorin for metastatic colorectal cancer. N Engl J Med.

[32] Johnson DH, Fehrenbacher L, Novotny WF, Herbst RS, Nemunaitis JJ, Jablons DM (2004). Randomized phase II trial comparing bevacizumab plus carboplatin and paclitaxel with carboplatin and paclitaxel alone in previously untreated locally advanced or metastatic non-small-cell lung cancer. J Clin Oncol.

[33] Bose P, Holter JL, Selby GB (2009). Bevacizumab in hereditary hemorrhagic telangiectasia. N Engl J Med.

[34] Iyer VN, Apala DR, Pannu BS, Kotecha A, Brinjikji W, Leise MD (2018). Intravenous bevacizumab for refractory hereditary hemorrhagic telangiectasia-related epistaxis and gastrointestinal bleeding. Mayo Clin Proc.

[35] Dupuis-Girod SGI, Saurin JC, Marion D, Guillot E, Decullier E, Roux A (2012). Bevacizumab in patients with hereditary hemorrhagic telangiectasia and severe hepatic vascular malformations and high cardiac output. JAMA.

[36] Vlachou PA, Colak E, Koculym A, Kirpalani A, Kim TK, Hirschfield GM (2013). Improvement of ischemic cholangiopathy in three patients with hereditary hemorrhagic telangiectasia following treatment with bevacizumab. J Hepatol.

[37] Mitchell A, Adams LA, MacQuillan G, Tibballs J, vanden Driesen R, Delriviere L. Bevacizumab reverses need for liver transplantation in hereditary hemorrhagic telangiectasia. Liver Transpl 2008;14:210–213. 10.1002/lt.21417 18236396

[38] Guilhem A, Fargeton AE, Simon AC, Duffau P, Harle JR, Lavigne C, et al. Intra-venous bevacizumab in hereditary hemorrhagic telangiectasia (HHT): a retrospective study of 46 patients. PLoS One. 2017;12. 10.1371/journal.pone.0188943. 10.1371/journal.pone.0188943 PMC5708634 29190827

[39] Palumbo A, Davies F, Kropff M, Bladé J, Delforge M, Leal da Costa F (2010). Consensus guidelines for the optimal management of adverse events in newly diagnosed, transplant-ineligible patients receiving melphalan and prednisone in combination with thalidomide (MPT) for the treatment of multiple myeloma. Ann Hematol.

[40] Cohen MH, Gootenberg J, Keegan P, Pazdur R (2007). FDA drug approval summary: bevacizumab (Avastin) plus carboplatin and paclitaxel as first-line treatment of advanced/metastatic recurrent nonsquamous non-small cell lung cancer. Oncologist.

[41] Li J, Zhou L, Chen X, Ba Y (2015). Addition of bevacizumab to chemotherapy in patients with ovarian cancer: a systematic review and meta-analysis of randomized trials. Clin Transl Oncol.

[42] Gressett SM, Shah SR (2009). Intricacies of bevacizumab-induced toxicities and their management. Ann Pharmacother.

[43] Sandler A, Gray R, Perry MC, Brahmer J, Schiller JH, Dowlati A (2006). Paclitaxel-carboplatin alone or with bevacizumab for non-small-cell lung cancer. N Engl J Med.

[44] Maestraggi Q, Bouattour M, Toquet S, Jaussaud R, Kianmanesh R, Durand F (2015). Bevacizumab to Treat Cholangiopathy in Hereditary Hemorrhagic Telangiectasia: Be Cautious: A Case Report. Medicine (Baltimore).

[45] Stickel F, Z'graggen K (2017). Bevacizumab for the treatment of Osler's disease – a note of caution. Liver Int.

[46] Muller YD, Oppliger R, Breguet R, Meyer P, Rubbia-Brandt L, Petignat PA (2016). Hereditary haemorrhagic telangiectasia: to transplant or not to transplant - is there a right time for liver transplantation?. Liver Int.

[47] European Reference Networks: https://ec.europa.eu/health/ern_en Accessed 26 Oct 2018.

[48] The European Reference Network for Vascular Diseases (VASCERN) HHT WG: https://vascern.eu/expertise/rare-diseases-wgs/hht-wg/ Accessed 26 Oct2018.

[49] The European Reference Network for Rare Multisystemic Vascular Diseases : https://vascern.eu/expertise/rare-diseases-wgs/hht-wg/ Accessed 26 Oct2018.

[50] Buscarini E, Botella LM, Geisthoff UW, Kjeldsen A, Mager HJ, Pagella F, et al. Vascern HHT survey 2: drug registry-part 1. Angiogenesis. 2017. 10.1007/s10456-017-9584-3.

[51] Buscarini E, Manfredi G, Gazzaniga P, Reduzzi L, Danesino C, Olivieri C (2009). Thalidomide for treatment of chronic severe bleeding in hereditary hemorrhagic telangiectasia. Hematol Meet Rep.

[52] CTCAE, 4.03 https://evs.nci.nih.gov/ftp1/CTCAE/CTCAE_4.03/. Accessed 15 Apr 2017.

[53] Buscarini E, Leandro G, Conte D, Danesino C, Daina E, Manfredi G (2011). Natural history and outcome of hepatic vascular malformations in a large cohort of patients with hereditary hemorrhagic teleangiectasia. Dig Dis Sci.

[54] EU European population, https://ec.europa.eu/eurostat/documents/2995521/9063738/3-10072018-BP-EN.pdf/ccdfc838-d909-4fd8-b3f9-db0d65ea457f. Accessed 28 Oct 2018.

[55] Shovlin CL, Bamford KB, Sabbà C, Mager HJ, Kjeldsen AD, Droege F, et al. Prevention of serious infections in hereditary haemorrhagic telangiectasia: roles for prophylactic antibiotics, the pulmonary capillaries- but not vaccination. Haematologica. 2018; in press. 10.3324/haematol.2018.209791 PMC6355489 30705116

[56] Livesey JA, Manning RA, Meek JH, Jackson JE, Kulinskaya E, Laffan MA (2012). Low serum iron levels are associated with elevated plasma levels of coagulation factor VIII and pulmonary emboli/deep venous thromboses in replicate cohorts of patients with hereditary haemorrhagic telangiectasia. Thorax.

[57] Thielemans L, Layton DM, Shovlin CL. Low serum haptoglobin and blood films suggest intravascular haemolysis contributes to severe anaemia in hereditary haemorrhagic telangiectasia. Haematologica. 2018. 10.3324/haematol.2018.205682 [Epub ahead of print]. 10.3324/haematol.2018.205682 PMC6442977 30337360

[58] Dupuis-Girod S, Buscarini E (2016). Hereditary hemorrhagic telangiectasia: to transplant or not to transplant?. Liver Int.

[59] Dupuis-Girod S, Buscarini E (2017). Response to bevacizumab for the treatment of Rendu-Osler disease-a note of caution. Liver Int.

[60] Shovlin CL, Buscarini E, Hughes JMB, Allison DJ, Jackson JE (2017). Long-term outcomes of patients with pulmonary arteriovenous malformations considered for lung transplantation, compared with similarly hypoxaemic cohorts. BMJ Open Respir Res.

[61] Devlin HL, Hosman AE, Shovlin CL (2013). Antiplatelet and anticoagulant agents in hereditary hemorrhagic telangiectasia. N Engl J Med.

[62] Shovlin CL, Sodhi V, McCarthy A, Lasjaunias P, Jackson JE, Sheppard MN (2008). Estimates of maternal risks of pregnancy for women with hereditary haemorrhagic telangiectasia (Osler-weber-Rendu syndrome): suggested approach for obstetric services. BJOG.

[63] Rizvi A, Macedo P, Babawale L, Tighe HC, Hughes JMB, Jackson JE (2017). Hemoglobin is a vital determinant of arterial oxygen content in hypoxemic patients with pulmonary arteriovenous malformations. Ann Am Thorac Soc.

[64] Kjeldsen AD, Kjeldsen J (2000). Gastrointestinal bleeding in patients with hereditary hemorrhagic telangiectasia. Am J Gastroenterol.

[65] Kjeldsen AD, Vase P, Green A (1999). Hereditary haemorrhagic telangiectasia: a population-based study of prevalence and mortality in Danish patients. J Intern Med.

[66] Kjeldsen A, Aagaard KS, Tørring PM, Möller S, Green A (2016). 20-year follow-up study of Danish HHT patients-survival and causes of death. Orphanet J Rare Dis.

[67] Brufsky AM, Hurvitz S, Perez E, Swamy R, Valero V, O'Neill V (2011). RIBBON-2: a randomized, double-blind, placebo-controlled, phase III trial evaluating the efficacy and safety of bevacizumab in combination with chemotherapy for second-line treatment of human epidermal growth factor receptor 2-negative metastatic breast cancer. J Clin Oncol.

[68] Hurwitz HI, Tebbutt NC, Kabbinavar F, Giantonio BJ, Guan ZZ, Mitchell L (2013). Efficacy and safety of bevacizumab in metastatic colorectal cancer: pooled analysis from seven randomized controlled trials. Oncologist.

[69] Chen X, Chen Y, Cai X, Zhang D, Fan L, Qiu H (2017). Efficacy and safety of bevacizumab in elderly patients with advanced colorectal cancer: a meta-analysis. J Cancer Res Ther.

[70] Misawa S, Sato Y, Katayama K, Nagashima K, Aoyagi R, Sekiguchi Y (2016). Safety and efficacy of thalidomide in patients with POEMS syndrome: a multicentre, randomised, double-blind, placebo-controlled trial. Lancet Neurol.

[71] Palumbo A, Waage A, Hulin C, Beksac M, Zweegman S, Gay F (2013). Safety of thalidomide in newly diagnosed elderly myeloma patients: a meta-analysis of data from individual patients in six randomized trials. Haematologica.

[72] Palmaro A, Rougé-Bugat ME, Gauthier M, Despas F, Moulis G, Lapeyre-Mestre M (2017). Real-life practices for preventing venous thromboembolism in multiple myeloma patients: a cohort study from the French health insurance database. Pharmacoepidemiol Drug Saf.

[73] Brinjikji W, Nasr DM, Wood CP, Iyer VN (2017). Pulmonary arteriovenous malformations are associated with silent brain infarcts in hereditary hemorrhagic telangiectasia patients. Cerebrovasc Dis.

