# Supplementary material for: Safety of thalidomide and bevacizumab in patients with hereditary hemorrhagic telangiectasia
Source: Orphanet J Rare Dis. 2019 Feb 4;14:28. doi: 10.1186/s13023-018-0982-4 (PMC6360670; doi:10.1186/s13023-018-0982-4)
Supplement: Supplementary file 1 — Questionnaire for VASCERN-HHT Survey Drug Registry- Part 1. (PDF 183 kb) [file 13023_2018_982_MOESM1_ESM.pdf]

Default Question Block

HHT SURVEY 2 - February 2017 - Drug Registry - Part 1

This survey is an activity of VASC–ERN , the European Reference Network on Rare Multisystemic Vascular Diseases

Elisabetta Buscarini, M.D.
Deputy Co-Chair of VASC-ERN/HHT
http://vascern.eu/expertise/rare-diseases-wgs/hht-wg/

VASCERN, the European Reference Network on Rare Multisystemic Vascular Diseases

Please, specify your identity.

I am a patient

I am a healthcare professional

I am a scientist

Have you used intravenous Bevacizumab (commercial name: Avastin)?

Yes

No

How did you find it? (Please rate it from 1 to 7, where 1=worst; 4=don't know; 7=best)

1	2	3	4	5	6	7

Have you used thalidomide?

Yes

No

How did you find it? (Please rate it from 1 to 7, where 1=worst; 4=don't know; 7=best)

1	2	3	4	5	6	7

Are you aware of any effects of intravenous Bevacizumab/Avastin in HHT patients?

Are you aware of any effects of thalidomide in HHT patients?

Have you personally prescribed or arranged intravenous Bevacizumab treatment for an HHT patient?

Yes

No

How many people have you prescribed it to?

<5

6-20

20-50

50-100

>100

Have any other patients prescribed intravenous Bevacizumab by other doctors reported their experience to you?

Yes

No

How many people?

<5

6-20

20-50

50-100

>100

Based on your own observations of patients you have seen, how much do you agree with the following statements:
(1=no agreement; 4=don't know; 7=full agreement)

	1	2	3	4	5	6	7
Intravenous Bevacizumab is helpful in treating HHT epistaxis	○	○	○	○	○	○	○

Intravenous Bevacizumab is helpful in treating HHT GI bleeding

Intravenous Bevacizumab is helpful in treating HHT hepatic VMs

Intravenous Bevacizumab is helpful in reducing transfusion dependency

Intravenous Bevacizumab is safe and has no significant side effects for people with HHT

Have you personally prescribed or arranged thalidomide treatment for an HHT patient?

Yes

No

How many people have you prescribed it to?

<5

6-20

20-50

50-100

>100

Have any other patients prescribed thalidomide by other doctors reported their experience to you?

Yes

No

How many people?

<5

6-20

20-50

50-100

>100

Based on your own observations of patients you have seen, how much do you agree with the following statements:
(1=no agreement; 4=don't know; 7=full agreement)

	1	2	3	4	5	6	7
Thalidomide is helpful in treating HHT epistaxis	○	○	○	○	○	○	○
Thalidomide is helpful in treating HHT GI bleeding	○	○	○	○	○	○	○
Thalidomide is helpful in treating HHT hepatic VMs	○	○	○	○	○	○	○
Thalidomide is helpful in reducing transfusion dependency	○	○	○	○	○	○	○
Thalidomide is safe and has no significant side effects for people with HHT	○	○	○	○	○	○	○

Would you like to be part of the VASCERN_HHT subcommittee evaluating the use of these agents further?

Yes

No

With intravenous Bevacizumab, have you seen any response(s) that you wondered if it/they might be a side effect (adverse event)?

Yes

Maybe

No

With thalidomide, have you seen any response(s) that you wondered if it/they might be a side effect (adverse event)?

Yes

Maybe

No

Powered by Qualtrics
