# Supplementary material for: Safety of thalidomide and bevacizumab in patients with hereditary hemorrhagic telangiectasia
Source: Orphanet J Rare Dis. 2019 Feb 4;14:28. doi: 10.1186/s13023-018-0982-4 (PMC6360670; doi:10.1186/s13023-018-0982-4)
Supplement: Supplementary file 2 — Questionnaire for VASCERN-HHT Survey Drug Registry- Part 2. (PDF 239 kb) [file 13023_2018_982_MOESM2_ESM.pdf]

Default Question Block

HHT SURVEY 2 - May 2017 - Drug Registry - Part 2

This survey is an activity of VASCERN , the European Reference Network on Rare Multisystemic Vascular Diseases

Thalidomide and Bevacizumab, have been increasingly used in the latest decade in patients with HHT. Their use in HHT is off-label; this aspect and their potential for adverse events warrant a great level of attention from scientific, clinical and lay HHT community.

This is why we have proposed the **VASCERN HHT SURVEY 2: Drug Registry; in Part 1** either patient or scientist or health care professional have been asked to summarize their experience, if any, with these drugs; their responses to Part 1 allowed to estimate number of patients so far treated and the opinions about these drugs.

Part 2 addresses health care professionals experience to capture adverse events occurred in patients treated with bevacizumab and thalidomide.

We ask you to fill the questionnaire by clicking the link below by **May 22, 2017.**

Results of this VASCERN survey will be reported at the International HHT Conference in Dubrovnik.

Thank you for taking the time of sharing your experience!

Elisabetta Buscarini, M.D. (Deputy CoChair, VASCERN-HHT)

Sophie Dupuis Girod, M.D. (CoChair VASCERN-HHT)

Claire L. Shovlin, Ph.D. F.R.C.P. (Chair VASCERN-HHT)

VASCERN, the European Reference Network
on Rare Multisystemic Vascular Diseases

HOW TO FILL THE QUESTIONNAIRE:

Please note that every time you leave the questionnaire without submitting, you can go back to the survey and edit it (please use the back button to modify your responses). Once you submit the questionnaire, it will

not be further modifiable.

You can also print the PDF of the questionnaire we sent you in the email to have an hard copy.

Please, specify your identity:

Name

Affiliation

Have you ever treated patients with **Bevacizumab**?

- Yes
- No

BEVACIZUMAB

Number of patients:

Number of females:

Mutation identified, number: ENG

Mutation identified, number: Alk1

Mutation identified, number: SMAD4

Sum of ages of all patients (e.g: 3 individuals 66, 75 ,67 yo= 208)

BEVACIZUMAB

DURATION OF TREATMENT

how many had only an induction cycle (6 administrations/every 3 weeks)?

how many had induction + maintenance?

sum of drug administrations in all patients having induction + maintenance

BEVACIZUMAB

DRUG DOSAGE

5 mg/kg: number of drug administrations

2.5 mg/kg: number of drug administrations

BEVACIZUMAB

ADMINISTRATION SCHEDULE

Every …(number) weeks for induction

Every …(number) weeks for maintenance

BEVACIZUMAB

INDICATION

For otherwise untreatable nose bleedings: number of patients

For otherwise untreatable GI bleedings: number of patients

Combination of both nose and GI bleeding: number of patients

For otherwise untreatable high output cardiopathy: number of patients

Have you ever treated patients with **Thalidomide**?

- Yes
- No

THALIDOMIDE

Number of patients:

Number of females:

Mutation identified, number: ENG

Mutation identified, number: Alk1

Mutation identified, number: SMAD4

Sum of ages of all patients (e.g: 3 individuals 66, 75 ,67 yo= 208)

THALIDOMIDE

DURATION OF TREATMENT

Sum of months of therapy of all patients

THALIDOMIDE

DAILY DRUG DOSAGE/patients' number

<50mg/.....

50 mg/…….

100 mg/…….

200 mg/…….

*mean dosage....../........

*for patients treated with different dosages during single treatment calculate average daily dosage

THALIDOMIDE

INDICATION

For otherwise untreatable nose bleedings: number of patients

For otherwise untreatable GI bleedings: number of patients

Combination of both nose and GI bleeding: number of patients

For otherwise untreatable high output cardiopathy: number of patients

ADVERSE EVENTS

An Adverse Event (AE) is any unfavorable and unintended sign (including an abnormal laboratory finding), symptom, or disease temporally associated with the use of a medical treatment or procedure that may or may not be considered related to the medical treatment or procedure.

Report every single AE (only if NOT present before the treatment) by answering to following questions; if you want to report more than one AE you will be re-directed every time to fill this section.

Block 1

Patient code (two first letters of Center name and patient number: example: CR1 for first patient from Crema center; if a patient had more than one AE fill a corresponding number of forms, but with the same assigned patient code)

Patient sex

- ◯ Male
- ◯ Female

HHT mutation:

- ◯ ENG
- ◯ Alk1
- ◯ SMAD4

Patient age

[ ]

Drug:

- ◯ Bevacizumab
- ◯ Thalidomide

Dosage:

Thalidomide.........mg/d	
Bevacizumab......mg/kg	
Total of Bevacizumab administrations	

Is the treatment ongoing?

- ◯ Yes
- ◯ No

On treatment from (months):

Treatment stopped since (months):

AE Type

- hypertension
- gastrointestinal perforation
- arterial thrombosis
- venous thrombosis/thromboembolic event
- Cardiac failure
- bleeding - if bleeding, specify site:

Bleeding

- cerebral
- pulmonary
- GI
- Other:……….

- Peripheral neuropathy
- Allergic reaction during bevacizumab infusion
- Joint pain
- Other:……………..

AE grade

Table to be made visible by click on: Table of AE grading

- 1
- 2
- 3

- ○ 4
- ○ 5

If death:

- ○ certainly related to drug
- ○ drug may have contributed
- ○ not related to drug
- ○ unknown cause

AE Outcome

- ○ resolved completely
- ○ resolved with sequelae
- ○ unresolved/worsened
- ○ unknown

Treatment interruption because of AE?

- ○ Yes
- ○ No

Improvement of AE after treatment interruption?

- ○ Yes
- ○ No

Treatment restarted?

- ○ Yes
- ○ No

Recurrence of AE after treatment restart?

- Yes
- No

Other concomitant drug possibly related to AE?

- ________ Yes (which one?)
- No

Any alternative cause identifiable for AE?

- ________ Yes (which one?)
- No

Do you want to insert another AE?

- Yes
- No and I want to submit my survey (Please note: once submitted the questionnaire will not be further modifiable)

Powered by Qualtrics
